# Radiomics Analysis of Multiparametric MRI for the Preoperative Prediction of Lymph Node Metastasis in Cervical Cancer

**DOI:** 10.3389/fonc.2020.01393

**Published:** 2020-08-20

**Authors:** Lina Hou, Wei Zhou, Jialiang Ren, Xiaosong Du, Lei Xin, Xin Zhao, Yanfen Cui, Ruiping Zhang

**Affiliations:** ^1^Department of Radiology, Shanxi Province Cancer Hospital, Shanxi Medical University, Taiyuan, China; ^2^Department of Radiology, Huzhou Central Hospital, Affiliated to Huzhou University, Huzhou, China; ^3^GE Healthcare China, Beijing, China; ^4^Department of Gynecology, Shanxi Province Cancer Hospital, Shanxi Medical University, Taiyuan, China

**Keywords:** nomograms, predictive value of tests, magnetic resonance imaging, uterine cervical neoplasms, lymph nodes

## Abstract

**Objective:** To develop and validate a radiomics predictive model based on multiparameter MR imaging features and clinical features to predict lymph node metastasis (LNM) in patients with cervical cancer.

**Material and Methods:** A total of 168 consecutive patients with cervical cancer from two centers were enrolled in our retrospective study. A total of 3,930 imaging features were extracted from T2-weighted (T2W), ADC, and contrast-enhanced T1-weighted (cT1W) images for each patient. Four-step procedures, mainly minimum redundancy maximum relevance (MRMR) and least absolute shrinkage and selection operator (LASSO) regression, were applied for feature selection and radiomics signature building in the training set from center I (*n* = 115). Combining clinical risk factors, a radiomics nomogram was then constructed. The models were then validated in the external validation set comprising 53 patients from center II. The predictive performance was determined by its calibration, discrimination, and clinical usefulness.

**Results:** The radiomics signature derived from the combination of T2W, ADC, and cT1W images, composed of six LN-status-related features, was significantly associated with LNM and showed better predictive performance than signatures derived from either of them alone in both sets. Encouragingly, the radiomics signature also showed good discrimination in the MRI-reported LN-negative subgroup, with AUC of 0.825 (95% CI: 0.732–0.919). The radiomics nomogram that incorporated radiomics signature and MRI-reported LN status also showed good calibration and discrimination in both sets, with AUCs of 0.865 (95% CI: 0.794–0.936) and 0.861 (95% CI: 0.733–0.990), respectively. Decision curve analysis confirmed its clinical usefulness.

**Conclusion:** The proposed MRI-based radiomics nomogram has good performance for predicting LN metastasis in cervical cancer and may be useful for improving clinical decision making.

## Introduction

Cervical cancer is the fourth most common cancer worldwide and ranks second as a cause of cancer-related death among women in developing countries, including China (1, 2). Lymph node metastasis (LNM) is one of the important determinants for prognosis and treatment planning (3, 4). Patients without LNM in early-stage cervical cancer show a high 5-year survival rate of 90%, while the 5-year survival rate rapidly deteriorated in patients with LNM, with only 65% (4, 5). Radical hysterectomy and pelvic lymph node dissection (PLND) are the conventional curative treatment options for stage IB–IIA cervical cancers, recommended by the International Federation of Gynecology and Obstetrics (FIGO) guidelines. However, approximately 10%−30% of patients with early-stage cervical cancer harbor LNM (6, 7). Adjuvant chemoradiotherapy is recommended for these patients with LNM diagnosed pathologically after surgery. Thus, a large proportion of patients might be over-treated and have to accept an unnecessary PLND, accompanied by increased adverse effects and more complications (8). Moreover, radical trachelectomy, an emerging fertility-sparing treatment for cervical cancer, was not eligible for patients with LNM (9). Therefore, accurate prediction of LNM is crucial for treatment strategy decision and predicting prognosis of patients with cervical cancer.

Magnetic resonance imaging (MRI) has long been the imaging modality for preoperative local staging and detection of LNM of cervical cancer in clinical practice, including T2-weighted (T2W), contrast-enhanced T1-weighted (cT1W) imaging, and diffusion-weighted imaging (DWI) (10, 11). These imaging methods have the potential to predict LNM; however, according to morphologic criteria such as size and shape, their efficacy in identifying LNM is unsatisfactory, especially that the sensitivity is relatively low (38%–56%) (12). The low sensitivity might be due to the existence of micrometastatic lymph nodes, which has consequently led to a considerable proportion of patients with cervical cancer being understaged (13, 14).

Radiomics, which involves the extraction of mineable high-dimensional imaging features from digital medical images, is gaining importance in personalized cancer therapy (15). This strategy has shown a great potential for improved diagnostic and prognostic in a wide range of cancer types (16–19). Few studies have suggested improvement in preoperative prediction of LNM by using different modalities-based radiomics analysis in cervical cancers (20–23). However, these studies might suffer from relatively small sample sizes, analysis of single sequence or single section rather than whole-tumor volume analysis, or lack of external validation.

Therefore, the aim of our two-center study was to develop and validate a multiparametric MRI-based radiomics model for the preoperative prediction of LNM in patients with cervical cancer.

## Materials and Methods

### Patients

Institutional ethics review board approval of all participating institutions was acquired for this two-center retrospective study, and the need for informed patient consent was waived.

**Figure 1:**
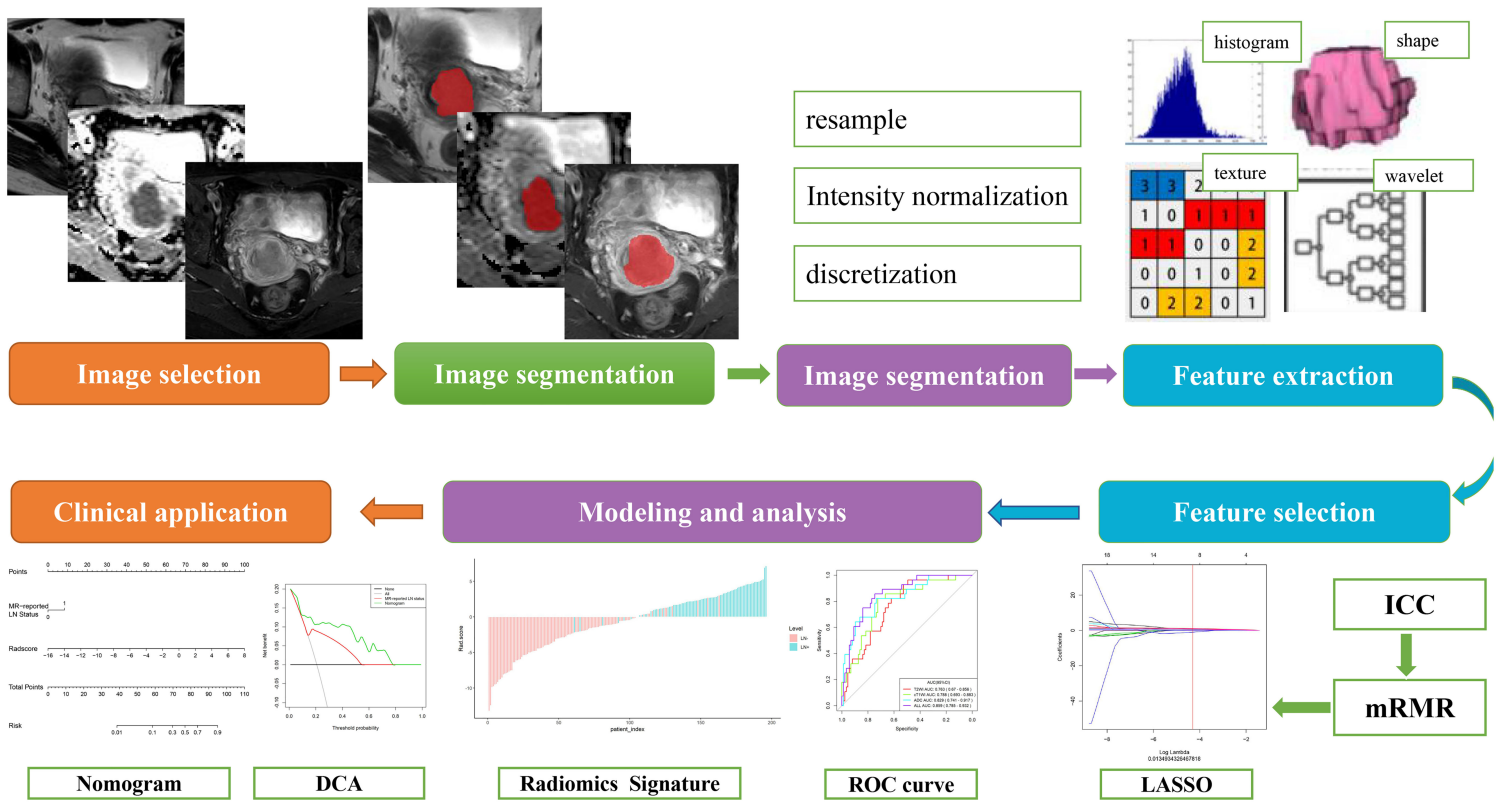
The workflow of radiomic analysis in the current study.

The inclusion criteria for patients included the following: (a) pathologically confirmed cervical squamous cell cancer (CSCC); (b) radical hysterectomy and pelvic lymphadenectomy performed; (c) without any prior treatment before surgical resection; (d) standard pelvic MRI performed 20 days before surgery; and (e) clinical and pathological characteristics were available. The exclusion criteria were as follows: (a) preoperative therapy (neoadjuvant chemotherapy, radiotherapy, or conization) performed; (b) lack of any MRI sequences, including T2WI without fat suppression, DWI, and cT1W MR imaging; (c) poor MR image quality resulting from motion artifacts; (d) lesions invisible on MRI sequences mentioned above. A total of 168 consecutive patients with cervical cancer from June 2012 to March 2016 were enrolled in our study. Among them, 115 patients from center I (Shanxi Province Tumor Hospital) were assigned as training set, while the 53 patients from center II (Huzhou Central Hospital affiliated to Zhejiang University School of Medicine) were used as the validation set. Figure 1 shows the workflow of radiomics analysis in the current study.

Baseline characteristics for all patients, including age, FIGO stage, and pathological LN status were derived from the medical records. MRI data, including the maximal tumor diameter (MTD) and the MR-reported LN status, were recorded by two radiologists with 12 and 8 years of experience in pelvic disease interpretation after reviewing all of the MRI scans. Note that those patients with the short diameter of largest LN larger than 10 mm were regarded as positive MR-reported LN status (12). Any disagreement was resolved by discussion and consensus.

### MRI Acquisition and Segmentation

Transverse T2WI without fat suppression, ADC images, as well as cT1W images were retrieved for radiomics feature extraction. The detailed information for MRI scan parameters was presented in Appendix E1.

The regions of interest (ROIs) were manually segmented along the border of the tumor on each slice of axial T2WI, DWI with a *b*-value of 800 s/mm^2^, as well as cT1W images, by using the ITK-SNAP 3.8 (www.itksnap.org), resulting in the volume of interest (VOI) for the three-dimensional whole tumor. For ADC maps, ROIs were placed on the region of high signal intensity on DW images with a *b* value of 800 s/mm^2^ firstly and then copied to the corresponding ADC maps, due to the higher resolution of DW images compared to ADC maps.

### Radiomics Feature Extraction and Reproducibility

Before the feature extraction, each MRI scan was normalized with *z*-score in order to obtain a standard normal distribution of the image intensities. Radiomics feature extraction was conducted using PyRadiomics (24). The gray level of each image was quantized to 25 gray levels. Afterward, 1,130 radiomics features were exacted for each sequence, including four categories: (a) first-order features, (b) shape-based features, (c) statistics-based textural features, and (d) wavelet and Laplacian of Gaussian (LoG) features. More information about these features and their reproducibility are presented in Appendix E2.

### Radiomics Feature Selection and Signature Construction

We devised a four-step procedure for reducing dimension and selecting robust features for each sequence of T2W, ADC, and cT1W, respectively. Firstly, inter-class correlation coefficients (ICCs) were used to assess the stability and reproducibility of radiomics feature extraction (Appendix E2). Secondly, minimum redundancy maximum relevance (MRMR) was performed to find a subset of both relevant and complementary features (25), and the top 20 features were selected. Thirdly, the least absolute shrinkage and selection operator (LASSO) logistic regression algorithm (26), with penalty parameter tuning conducted by 10-fold cross-validation, was then applied to select LN-status-related features with non-zero coefficients. Finally, backward elimination was added to reduce the number of remaining final features. For the combination of the above three sequences, all the selected key features of each sequence were combined and were introduced to the multivariate logistic regression to build the radiomics signature. Backward stepwise selection was applied with Akaike's information criterion (AIC) as the stopping rule.

The predictive accuracy of the radiomics signature was assessed by the area under the receiver operator characteristic (ROC) curve (AUC) in both the training and validation sets. The corresponding sensitivity, specificity, and accuracy were calculated. Moreover, discrimination of the radiomics signature in the MRI-reported LN-negative subgroup was also evaluated using the AUC in the whole set.

### Development, Performance, and Validation of Radiomics Nomogram

Similarly, the radiomics signature and all mentioned clinical candidate predictors were tested in the stepwise multivariate logistic regression model to develop a radiomics nomogram for predicting LNM in the training set, also with AIC as the stopping rule. To provide a more understandable outcome measure, a radiomics nomogram was then constructed by the selected predictors.

The discrimination performance of established models was quantified by the AUCs. The AUCs of models were compared using a DeLong test (27). The calibration of the radiomics nomogram was assessed with a calibration curve, by plotting via bootstrapping with 1,000 resamples, and the goodness of fit was assessed with the Hosmer–Lemeshow test (28). The performance of the radiomics nomogram was then tested in the validation set by using the formula derived from the training set.

### Clinical Use

To determine the clinical usefulness of the radiomics nomogram and MRI-reported LN status, a decision curve analysis (DCA) was performed by calculating the net benefits at different threshold probabilities in the validation sets (29).

### Statistical Analysis

Continuous variables were compared by using the Student *t* test or Mann–Whitney *U*-test, and categorical variables were compared by using Chi-Squared or Fisher's exact test, as appropriate. All statistical analyses were conducted with R 3.6.0 (http://www.r-project.org) and MedCalc 15.8 (MedCalc, Mariakerke, Belgium). The packages in R software are described in Appendix E3. A two-sided *P*-value of less than 0.05 was considered significant.

## Results

### Patient Characteristics

Patient characteristics from two centers were summarized in Table 1 and Table S1. The rates of LNM in the training and validation sets remain balanced (24.3% and 20.8%, respectively, *P* = 0.608), as well as age, clinical stage, MTD, and MR-reported LN status (*P* = 0.209–0.896). Additionally, none of the above clinical characteristics differed significantly between the LN metastasis and LN-negative groups (*P* = 0.055–0.845), except for the MTD in the validation set (*P* = 0.030; Table 1). According to the subjective MRI-reported LN status, 48.7% (19/39) patients with LNM were understaged and 13.2% (17/129) of patients without LNM were overstaged in the whole set. The overall accuracy of the subjective evaluation was 78.6% (132/168), and sensitivity and specificity were 51.3 and 86.8%, respectively.

**Table 1:** Demographic and clinical characteristics of patients with LARC in the training and validation sets.

**Characteristics**	**Training set**			**Validation set**		***P*-value**
	**LN(+) (*n* = 28)**	**LN(–) (*n* = 87)**		**LN(+) (*n* = 11)**	**LN(–) (*n* = 42)**	
Age, mean ± SD, years	49.86 ± 7.68	52.10 ± 9.87	0.216	52.36 ± 7.89	53.14 ± 12.44	0.845
Clinical stage			0.827			0.812
FIGO IB	21 (75.0%)	67 (77.0%)		8 (72.7%)	32 (76.2%)	
FIGO IIA	7 (25.0%)	20 (23.0%)		3 (27.3%)	10 (23.8%)	
MTD			0.055			0.030
≤ 4 cm	13 (46.4%)	58 (66.7%)		5 (45.5%)	33 (78.6%)	
>4 cm	15 (53.6%)	29 (33.3%)		6 (54.5%)	9 (21.4%)	
MRI-reported LN status			0.000			0.005
Positive	14 (50.0%)	11 (12.6%)		6 (54.5%)	6 (14.3%)	
Negative	14 (50.0%)	76 (87.4%)		5 (45.5%)	36 (85.7%)	
Median Rad-score^*^	0.688 (−0.056 to 1.411)	−1.039 (−2.056 to −0.276)	0.000	0.191 (−0.370 to 1.1721)	−1.108 (−2.359 to −0.364)	0.001

*Data are number of patients; data in parentheses are percentage unless otherwise indicated. FIGO, International Federation of Gynecology and Obstetrics; LN, lymph node; MTD, the MRI-reported maximal tumor diameter*.

* *Data in parentheses are interquartile range*.

### Feature Selection and Radiomics Signature Construction

In total, 3,930 radiomics features were extracted for each patient. From these features, we selected 1,203, 1,169, and 1,167 features with high stability and reproducibility (both intra-observer and inter-observer ICCs > 0.80) for T2W, ADC, and cT1W images, respectively. After the MRMR algorithm was applied, 20 features remained for each sequence and were subjected to further selection by the LASSO method and backward elimination. Among them, the final five, seven, and four remaining features were selected for T2W, ADC, and cT1W sequence, respectively. These selected features could be found in the Rad-score calculation formula of each modality presented in Appendix E4.

**Figure 2:**
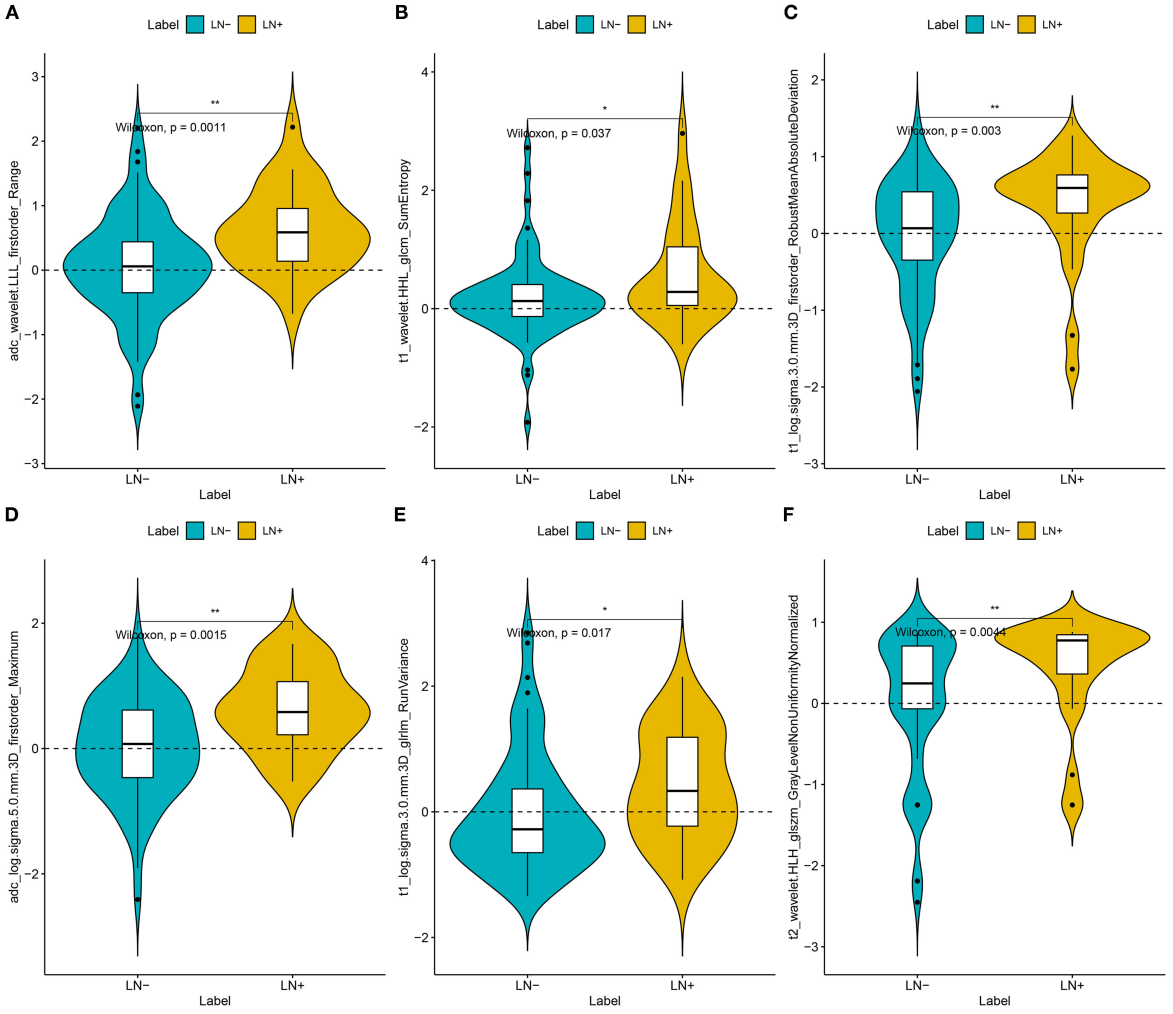
Plots **(A–F)** present the boxplots of the six radiomics feature with significant difference between the LN metastasis (LN+) and LN negative (LN–) groups in the training datasets, respectively.

**Figure 3:**
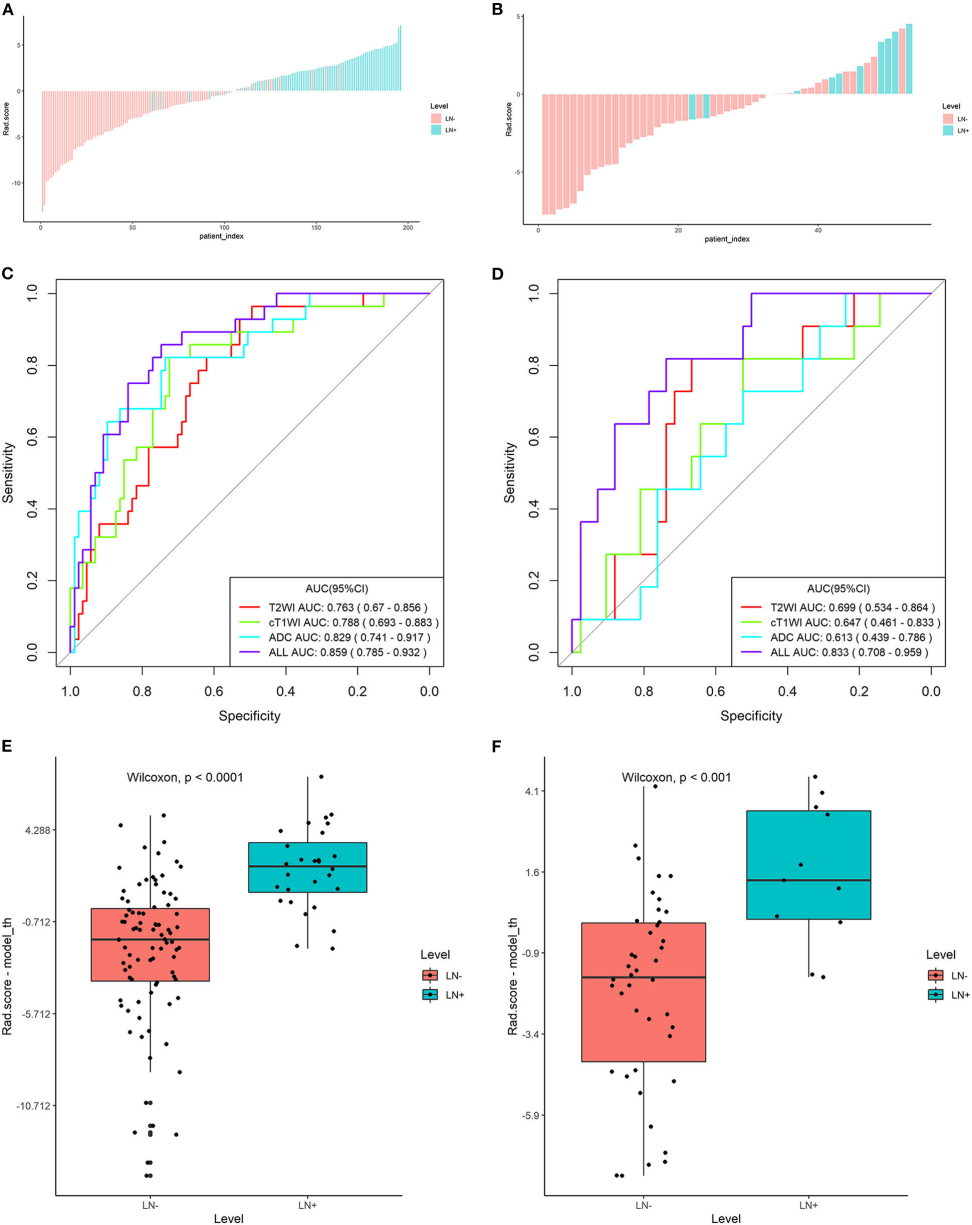
Plots **(A,B)** show the Rad-score for each patient, plots **(C,D)** show the receiver operating characteristic (ROC) curves of the radiomics signature derived from T2W, ADC, and cT1W images and their combination, and plots **(E,F)** present the boxplots of the Rad-score in the training and validation sets, respectively.

For the radiomics signature from the combination of all above sequences, 16 radiomics features were reduced to six features after the multivariate logistic regression analysis. All these six features were significantly different between patients with and those without LNM (all *P* < 0.05; Figure 2). The corresponding radiomics signature was constructed, with Rad-score calculated, according to the following formula: Rad-score = −2.0561 + 1.4492 × ADC_wavelet-LLL_firstorder_Range + 1.2371 × cT1WI_wavelet-HHL_glcm_SumEntropy – 1.1219 × cT1WI_loG_3.0_firstorder_RobustMeanAbsoluteDeviation + 0.8637 × ADC_loG_5.0_firstorder_Maximum + 0.5790 × cT1WI_loG_3.0_glrlm_RunVariance – 1.2105 × T2_wavelet-HLH_glszm_GrayLevelNonUniformityNormalized. Rad-score for each patient in the training and validation sets was shown in Figures 3A,B.

### Validation of Radiomics Signature

The radiomics signature derived from T2W images yielded AUCs of 0.763 [95% confidence interval (CI): 0.670–0.856] and 0.699 (95% CI: 0.534–0.864) in the training and validation sets, showing favorable predictive efficacy. Similarly, AUCs of 0.829 (95% CI: 0.741–0.917) and 0.788 (95% CI: 0.693–0.883) were acquired from the radiomics signature derived from ADC and cT1W images in the training set and then confirmed in the validation set with the AUCs of 0.613 (95% CI: 0.439–0.786) and 0.647 (95% CI: 0.461–0.833), respectively (Figures 3C,D).

The Rad-score derived from joint T2W, ADC, and cT1W images was significantly higher in patients with LNM than those without LNM in the training set (median, 0.688 *vs* −1.039; *P* < 0.000) and then confirmed in the validation set (median, 0.191 *vs* −0.108; *P* < 0.001) (Table 1; Figures 3E,F). The radiomics signature from the above sets yielded the highest AUC of 0.859 (95% CI: 0.785–0.932) and 0.833 (95% CI: 0.708–0.959) in the training and validation sets, respectively, suggesting that the radiomics signatures from the joint three modalities achieved better predictive efficacy than Rad-score from either of them alone (Figures 3C,D). The sensitivities were high, with 85.7 and 81.8% in the two sets, respectively, which was significantly higher than the subjective evaluation. Details regarding the performance of radiomics signature are shown in Table 2.

**Table 2:** Performance of the radiomics signature and nomogram.

**Metrics**	**Radiomics signature**		**Radiomics nomogram**	
	**Training dataset**	**Validation dataset**	**Training dataset**	**Validation dataset**
AUC (95%)	0.859 (0.785–0.932)	0.833 (0.708–0.959)	0.865 (0.794–0.936)	0.861 (0.733–0.99)
Accuracy (95%)	0.774 (0.687–0.847)	0.755 (0.617–0.862)	0.757 (0.668–0.832)	0.868 (0.747–0.945)
Sensitivity (95%)	0.857 (0.643–0.964)	0.818 (0.455–1.000)	0.929 (0.714–1.000)	0.818 (0.545–1.000)
Specificity (95%)	0.747 (0.448–0.874)	0.738 (0.405–0.952)	0.701 (0.368–0.839)	0.738 (0.381–0.976)

*AUC, area under ROC curve*.

**Figure 4:**
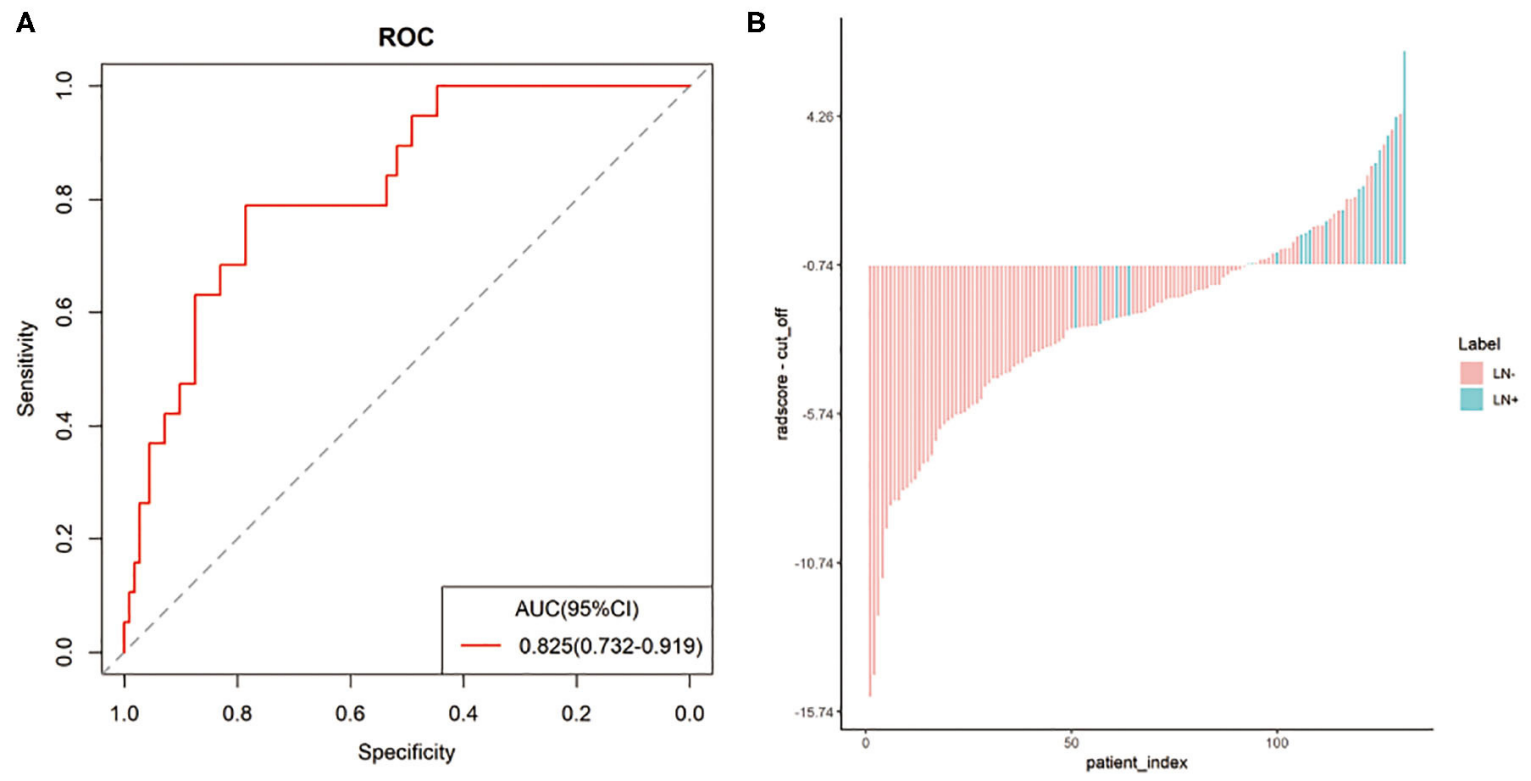
The predictive performance of the radiomics signature in the MRI-reported LN-negative subgroup. Plots **(A,B)** show the ROC curve of the radiomics signature and the Rad-score of individual patients in the MRI-reported LN-negative subgroup.

In the entire sets, significant association between the Rad-score derived from all three modalities and pathological LN status was observed when stratified analysis was performed (Table S2). In addition, in the MRI-reported LN-negative subgroup, 14.5% (19/131) of patients were understaged. Encouragingly, the radiomics signature also showed good discriminatory in this subgroup, with AUC of 0.825(95% CI: 0.732–0.919; Figure 4A). The radiomics-based risk classifier also achieved a diagnostic accuracy of 81.7% (117 of 131). Among them, most of the patients with pathological LN metastasis (78.9%, 15/19) would avoid being understaged by using the cutoff value of the radiomics signature (−0.74) (Figure 4B).

### Development, Performance, and Validation of Radiomics Nomogram

**Figure 5:**
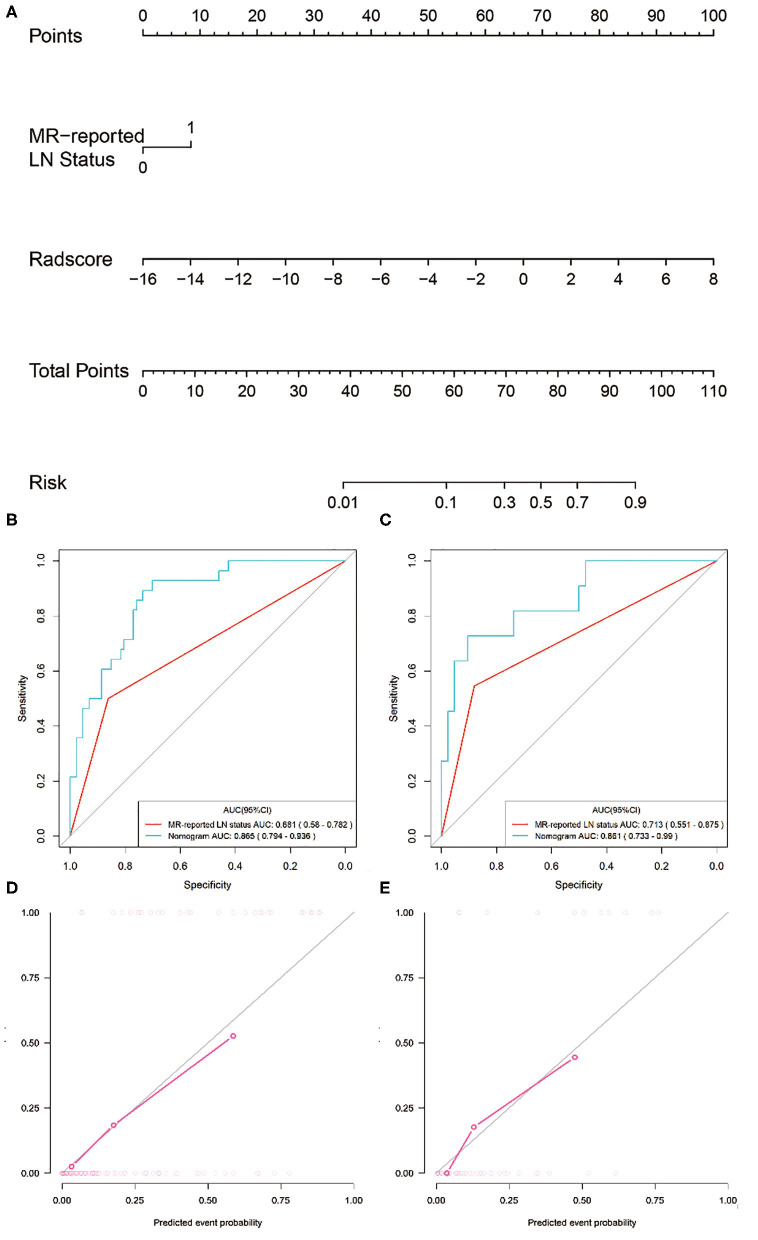
Radiomics nomogram developed with ROC curves and calibration curves. **(A)** A radiomics nomogram was developed for the prediction of LNM in the training set, with radiomics signature and MRI-reported LN status incorporated. Comparison of ROC curves between the radiomics nomogram and MRI-reported LN status alone for the prediction of LN metastasis in the **(B)** training and **(C)** validation sets. Plots **(D,E)** present the calibration curves of the radiomics nomogram in the training and validation sets, respectively.

Two variables, including MRI-reported LN status and the radiomics signature, were identified as independent predictors for predicting LNM based on the multivariate logistic regression analysis (Table S3). A radiomics nomogram, incorporating the above independent predictors, was then constructed (Figure 5A).

All ROC curves were provided in Figures 5B,C. The radiomics nomogram showed the highest discrimination ability for predicting LNM, with an AUC of 0.865 (95% CI: 0.794–0.936), significantly higher than that of MRI-reported LN status alone [AUC, 0.681 (95% CI: 0.580–0.782); *P* < 0.001]. Similarly, the radiomics nomogram yielded the greatest AUC of 0.861 (95% CI: 0.733–0.990) in the validation set, confirming that the radiomics nomogram achieved better predictive efficacy than MRI-reported LN status alone [AUC, 0.713 (95% CI: 0.551–0.875); *P* = 0.04].

Figure 5D illustrates the calibration curve of the radiomics nomogram, with good agreement between predicted and observed LN metastasis in the training set. The Hosmer–Lemeshow test yielded a non-significant *P-*value of 0.23, suggesting no departure from the perfect fit. The favorable calibration of the radiomics nomogram was further confirmed in the validation set (Figure 5E), with the *P*-value of 0.55 for the Hosmer–Lemeshow test.

### Clinical Use

**Figure 6:**
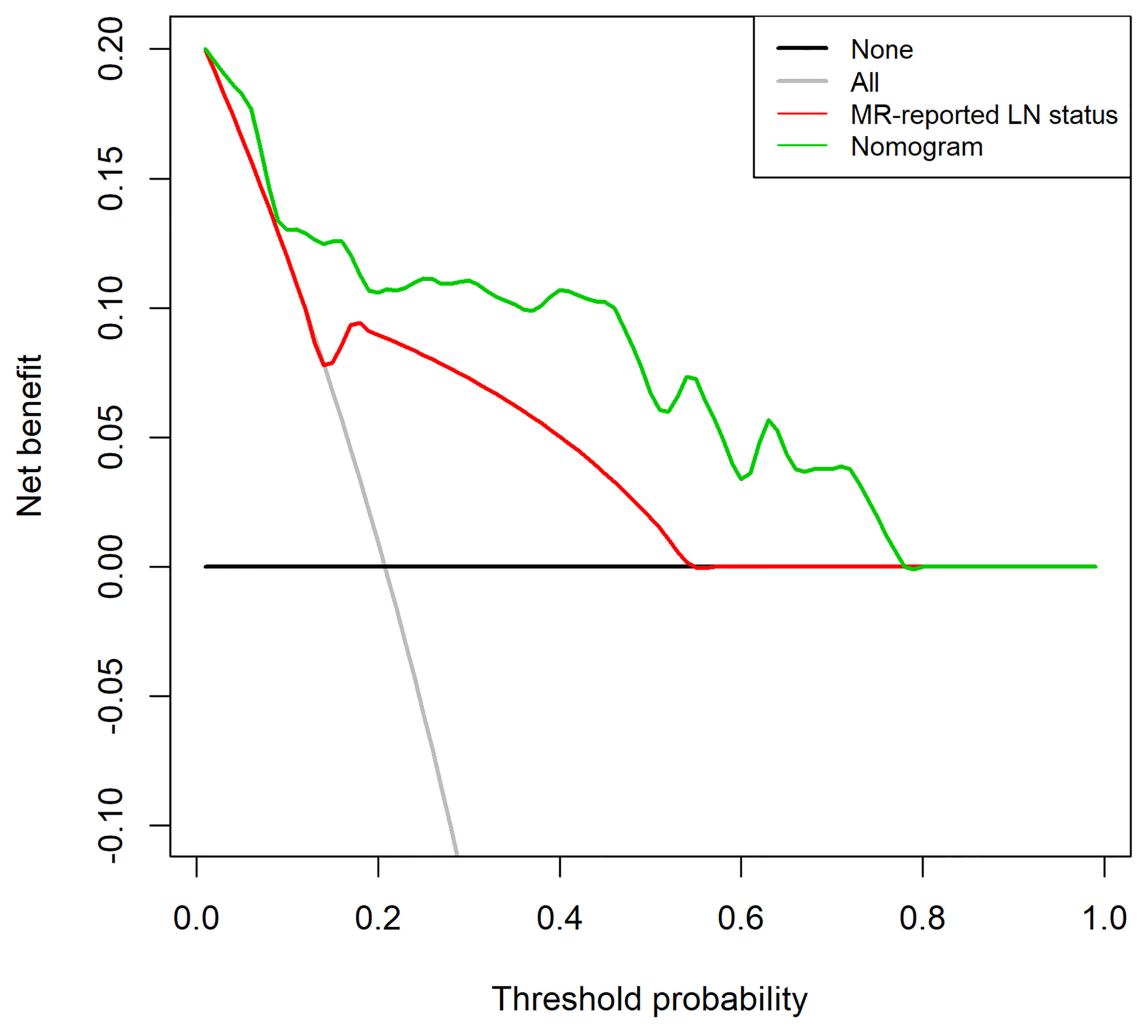
Decision curve analysis (DCA) for the radiomics nomogram and the MRI-reported LN status in the validation set. The *y*-axis represents the net benefit. The *x*-axis represents the threshold probability. The decision curves showed that if the threshold probability is over 10%, the application of radiomics nomogram to predict LNM adds more benefit than treating all or none of the patients and MRI-reported LN status.

The DCA for the radiomics nomogram and MRI-reported LN status were presented in Figure 6. The DCA showed that if the threshold probability was more than 10%, using the radiomics nomogram to predict LNM provided a better net benefit than treat-all-patients scheme or the treat-none scheme, as well as the MRI-reported LN status, indicating that the nomogram was clinically useful.

## Discussion

In our study, we successfully developed a radiomics-based nomogram incorporating the multiparametric MRI-based radiomics signature and the MRI-reported LN status for individualized prediction of LNM in patients with cervical cancer before surgery, and its findings were also validated in the external validation set. The proposed radiomics nomogram demonstrated favorable discrimination in both sets, outperforming the subjective MRI-reported LN status. Promisingly, in the MRI-reported LN-negative subgroup, the radiomics signature also showed favorable discriminative ability.

The accurate detection LNM of using visual judgment (conventional MRI) remains challenging in clinical settings. Our results showed that a considerable proportion of patients were misclassified according to the morphological evaluation on MR images, especially with very low sensitivity (51.3%), which might be due to false negatives caused by small LN metastasis, consistent with several previous studies (12). Although pelvic sentinel lymph node biopsy provides a valuable means for detecting LNM, it is still invasive and limited to the detection of small LN metastasis. Therefore, it is necessary to develop a non-invasive and reliable predictive tool for the prediction of LNM in patients with cervical cancer.

Radiomics hypothesizes that the intratumor heterogeneity, which was difficult to detect visually, could be exhibited on the spatial distribution of voxel intensities. To develop the radiomics signature, the 3,930 candidate radiomics features were reduced to only six predictors. Interestingly, wavelet (3/6) and LoG features (3/6) each account for half in that used in our optimal radiomics signature. The LoG filter extracts discriminative texture patterns from multiple space scale and could smooth images and increase the efficiency of capturing phenotypic features related to tumoral heterogeneity. Similarly, the wavelet features could reflect multi-frequency information at different scales unrecognized by the naked eye to quantify tumor heterogeneity. All these high-dimensional features were significantly higher in the LNM group in our study. It also has been proven that LoG filtering and wavelet translation were the important components in building radiomics signatures by several MRI-based radiomics studies (30–32).

The radiomics signature developed in our study showed favorable discrimination for predicting LNM in the training and external validation sets, with AUCs of 0.859 and 0.833, respectively. The performance of our radiomics signature was comparable to previous studies (22, 30). Kan et al. (22) reported that the SVM-based radiomics signatures derived from T2W and cT1W images were associated with LNM, with an AUC of 0.753 in the primary cohort. Wu et al. (30) found that radiomics signatures based on multiparametric MRI, especially functional map derived from ADC and dynamic contrast enhanced (DCE) MRI, was useful for predicting LNM with AUCs ranged from 0.747 to 0.850. However, the absence of external validation or technical differences between quantitative sequences of these studies limit their clinical applicability.

Encouragingly, the radiomics signature also showed good discriminatory in the MRI-reported LN-negative subgroup with an AUC of 0.825. The false-negative LN status might result in the chosen surgery and unnecessary PLND as their first treatment choice, with the following adjuvant chemoradiotherapy and more severe complications thereafter. Consequently, the false-negative rate of subjective MRI evaluation should be avoided as much as possible. Benefiting from the relatively high sensitivity of our radiomics signature, 15 out of 19 patients with pathological LNM were accurately diagnosed and might convert the treatment to radical chemoradiotherapy rather than surgery.

Our results also demonstrated that the radiomics signature from joint T2W, ADC, and cT1W images performed better than those from either of them alone in predicting LNM in cervical cancer. These sequences reflected different aspects of tumor, such as tumor intensity, cellularity, and vascularization; thus, the combination of these sequences could take full advantages of them and reflect much more comprehensive information about the tumors. Some studies indicated that some histogram or texture parameters derived from ADC or dual-energy CT images could predict the LN status in cervical cancer (33–35). This has been confirmed by our present study that the Rad-score derived from T2W, ADC, or cT1W images was significantly higher in the LNM group. That is, the features of LNM presented higher textual pattern complexity or heterogeneity than those of LN negative.

The clinical relevance of our study lies in providing an easy-to-use tool, the radiomics nomogram, for clinicians. Our study supported that the radiomics nomogram integrating the radiomics signature and MRI-reported LN status could achieve greater predictive efficacy than the subjective MRI evaluation alone, with a higher AUC and better calibration, consistent with previous studies (20, 23). Nevertheless, some notes should be emphasized. First, different clinical risk factors might be identified as independent predictors for predicting LNM, in spite of most of them in the multivariable logistic model were minuscule compared with the radiomics signatures. These results might indicate that some clinical features could serve as a biomarker for the prediction of LNM in cervical cancer, whereas the radiomics signature could generate more potentially relevant and informative metrics than semantic phenotypic features. Secondly, whole-tumor VOIs, rather than signal slice ROIs, could provide a robust way to characterize the heterogeneity of the entire lesion. Lastly, our models were validated in the external set with good calibration, and the DCA also confirmed its clinical usefulness.

Our study had several limitations. First, prospective study from more centers with considerably large cohorts are needed to further confirm the performance of our radiomics nomogram. Second, all the subjects in our study were CSCC. Different types of cervical cancer will be thoroughly studied in the future. Furthermore, genomic features, such as vascular endothelial growth factor (VEGF) expression, should be investigated and incorporated into the predictive model (21).

In conclusion, our two-center study developed and validated a multiparametric MRI-based radiomics model, incorporating the radiomics signature and the MRI-reported LN status, to facilitate preoperative evaluation of LN status in patients with cervical cancer, and thus providing a non-invasive and convenient tool to guide individual treatment strategies for those patients.

## Data Availability Statement

The raw data supporting the conclusions of this article will be made available from the corresponding author on reasonable request.

## Ethics Statement

The studies involving human participants were reviewed and approved by the Shanxi Province Cancer Hospital Ethics Committee and Huzhou Central Hospital Ethics Committee. Written informed consent for participation was not required for this study in accordance with the national legislation and the institutional requirements.

## Author Contributions

LH, WZ, YC, and RZ: conception and design. LH, WZ, XZ, and JR: development of methodology (acquired and managed patients, provided facilities, etc.). WZ, JR, and YC: analysis and interpretation of data (e.g., statistical analysis, biostatistics, and computational analysis). LH, YC, and RZ: writing, review, and/or revision of the manuscript. RZ, JR, XD, LX, and RZ: administrative, technical, or material support (i.e., reporting or organizing data, constructing databases). All authors contributed to the article and approved the submitted version.

## Conflict of Interest

JR was employed by the company GE Healthcare. The remaining authors declare that the research was conducted in the absence of any commercial or financial relationships that could be construed as a potential conflict of interest.

## Abbreviations

LNM: lymph node metastasis
PLND: pelvic lymph node dissection
FIGO: International Federation of Gynecology and Obstetrics
MRI: magnetic resonance imaging
CSCC: cervical squamous cell cancer
DWI: diffusion-weighted imaging
FOV: field of view
ADC: apparent diffusion coefficient
ROI: region of interest
GLCM: gray-level co-occurrence matrix
GLRLM: gray-level run length matrix
GLSZM: gray-level size zone matrix
NGTDM: neighboring gray tone difference matrix
GLDM: gray-level dependence matrix
LoG: Laplacian of Gaussian
ICC: interclass correlation coefficient
MRMR: minimum redundancy maximum relevance
LASSO: least absolute shrinkage and selection operator
ROC: receiver operating characteristic
DCA: decision curve analysis.
